# ATP-degrading ENPP1 is required for survival (or persistence) of long-lived plasma cells

**DOI:** 10.1038/s41598-017-18028-z

**Published:** 2017-12-19

**Authors:** Hongsheng Wang, Ines Gonzalez-Garcia, Javier Traba, Shweta Jain, Solomon Conteh, Dong-Mi Shin, Chenfeng Qi, Yuanyuan Gao, Jiafang Sun, Sungyun Kang, Sadia Abbasi, Zohreh Naghashfar, Jeongheon Yoon, Wendy DuBois, Alexander L. Kovalchuk, Michael N. Sack, Patrick Duffy, Herbert C. Morse

**Affiliations:** 1 Laboratory of Immunogenetics, National Institute of Allergy and Infectious Diseases, National Institutes of Health, Rockville, MD 20852 USA; 2 Laboratory of Mitochondrial Biology and Metabolism, National Heart, Lung and Blood Institute, National Institutes of Health, Bethesda, MD 20814 USA; 3 Laboratory of Malaria Immunology and Vaccinology, National Institute of Allergy and Infectious Diseases, National Institutes of Health, Rockville, MD 20852 USA; 4 Department of Food and Nutrition, Seoul National University, Seoul, 151-742 Korea; 5 Laboratory of Cancer Biology and Genetics, National Cancer Institute, National Institutes of Health, Bethesda, MD 20814 USA; 6 Present Address: Celgene Institute of Translational Research Europe, Parque Tecnológico Cartuja 93, c/Isaac Newton n.4, E-41092 Seville, Spain; 7 Present Address: Department of Medicine, Uniformed Services University of the Health Sciences, Bethesda, MD 20814 USA; 8 Present Address: Department of Biology, Indiana University, Myers Hall 230, 915 E. 3rd St., Bloomington, IN 47405 USA

## Abstract

Survival of antibody-secreting plasma cells (PCs) is vital for sustained antibody production. However, it remains poorly understood how long-lived PCs (LLPCs) are generated and maintained. Here we report that ectonucleotide pyrophosphatase/phosphodiesterase 1 (ENPP1) is preferentially upregulated in bone marrow LLPCs compared with their splenic short-lived counterparts (SLPCs). We studied ENPP1-deficient mice (*Enpp1* ^*−/−*^) to determine how the enzyme affects PC biology. Although *Enpp1* ^*−/−*^ mice generated normal levels of germinal center B cells and plasmablasts in periphery, they produced significantly reduced numbers of LLPCs following immunization with T-dependent antigens or infection with plasmodium *C*. *chabaudi*. Bone marrow chimeric mice showed B cell intrinsic effect of ENPP1 selectively on generation of bone marrow as well as splenic LLPCs. Moreover, *Enpp1* ^*−/−*^ PCs took up less glucose and had lower levels of glycolysis than those of wild-type controls. Thus, ENPP1 deficiency confers an energetic disadvantage to PCs for long-term survival and antibody production.

## Introduction

B cells undergo terminal differentiation upon stimulation with T-dependent or T-independent antigens. There are three fates of a stimulated B cell: differentiation into a memory B cell, a PC, or death by apoptosis. It has been demonstrated that PC can be generated by either extrafollicular or germinal center (GC) pathways in spleens and lymph nodes. While most PCs are thought to live only several days^1–4^, some manage to survive for long periods of time, sometimes for years, at particular anatomical sites such as the bone marrow (BM)^5,6^. These long-lived PCs (LLPCs) contribute to prolonged and sustained protection from re-infection (beneficial) or to long-term supply of self-damaging autoantibodies (pathogenic). Enhancing protective vaccine-induced LLPCs, to malaria, for example, and dampening pathogenic autoreactive LLPCs, such as those contributing to systemic lupus erythematosus, have been major hurdles in managing both diseases.

How LLPCs are generated and maintained in the BM is incompletely understood. It is thought that support for LLPC survival is mediated by cells in BM niches, including reticular stromal cells^7,8^, osteocytes^9^, megakaryocytes^10^, basophils^11^, and eosinophils^12^. These diverse cells provide crucial signals to LLPCs through direct cell-cell contact and/or the secretion of soluble factors such as IL-6 and APRIL^7,13–15^. Unlike long-lived hematopoietic stem cells (HSC), which also are resting cells and occupy similar BM niches, LLPCs are resting but metabolically active given the fact that a single PC can produce antibodies at up to 10^3^ molecules per second^16^. How LLPCs are programed to be metabolically distinct from other B cell types has remained unknown until recently. Lam *et al*. elegantly demonstrated that LLPCs have an advantage over SLPCs in the use of glucose and mitochondrial import of pyruvate for survival and antibody production^17^. Because metabolic pathways are regulated by a diverse array of signals stimulated by environmental cues, it remains unclear what cell surface receptors and what signals are responsible for the metabolic programing of LLPC. In this report, we identified ENPP1 as a novel membrane enzyme receptor critical for LLPC survival by regulating glucose uptake and energy production.

ENPP1 was discovered in 1970 by Takahashi, Old and Boyse as a membrane alloantigen restricted primarily to plasmacytomas and normal plasma cells (PCs) that they termed plasma cell alloantigen 1 (PC-1)^18^. ENPP1 is expressed in many non-lymphoid tissues including cartilage, heart, kidney, liver, and salivary gland, and is highly expressed in chondrocytes, osteoblasts, and vascular smooth muscle cells^19,20^. The function of ENPP1 is multifaceted. First, ENPP1 has broad substrate specificity, including ATP, UTP, cAMP, and 2′3′-cGAMP^21–24^. The enzyme catalyzes 5′-phosphodiesterase bonds, mostly in ATP, to generate nucleoside 5′-monophosphates and inorganic pyrophosphate (PPi)^21,24^, the latter being an inhibitor of mineral crystallization during the process of bone formation. Consistent with this activity, mice with inactivating mutant alleles of *Enpp1* ^25,26^ or a genetically engineered null allele^27^ exhibit “stiff joints” and “tiptoe walking” due to excessive calcification of joints and paraspinal ligaments. Mutations of *Enpp1* also cause blood vessel calcification in both mice^25,28^ and humans^29–32^. In addition, PPi is a stable high energy compound and can substitute for an ATP-derived energy supply at least in *Entamoeba histolytica* ^33^. Second, ENPP1 mediates nucleotide recycling by breaking down ATP to AMP, which is then converted to adenosine by 5′ nucleotidase^21^. Adenosine is then transported freely into cells for metabolism. Both ATP and adenosine are known to have immunoregulatory functions^34^. Third, ENPP1 is involved in adipocyte differentiation^35^ and plays a role in carbohydrate metabolism and insulin resistance (reviewed in^36^).

Although expression of ENPP1 on PCs was recognized almost five decades ago, little is known about the function of this molecule in PCs. In this report, we investigated the functions of ENPP1 in PC biology using *Enpp1* ^*−/−*^ mice. Our data demonstrate that while ENPP1 is dispensable for normal B cell development, it is essential for the development and survival of LLPCs.

## Results

### Expression of ENPP1 gradually increases during B cell and PC maturation

**Figure 1:**
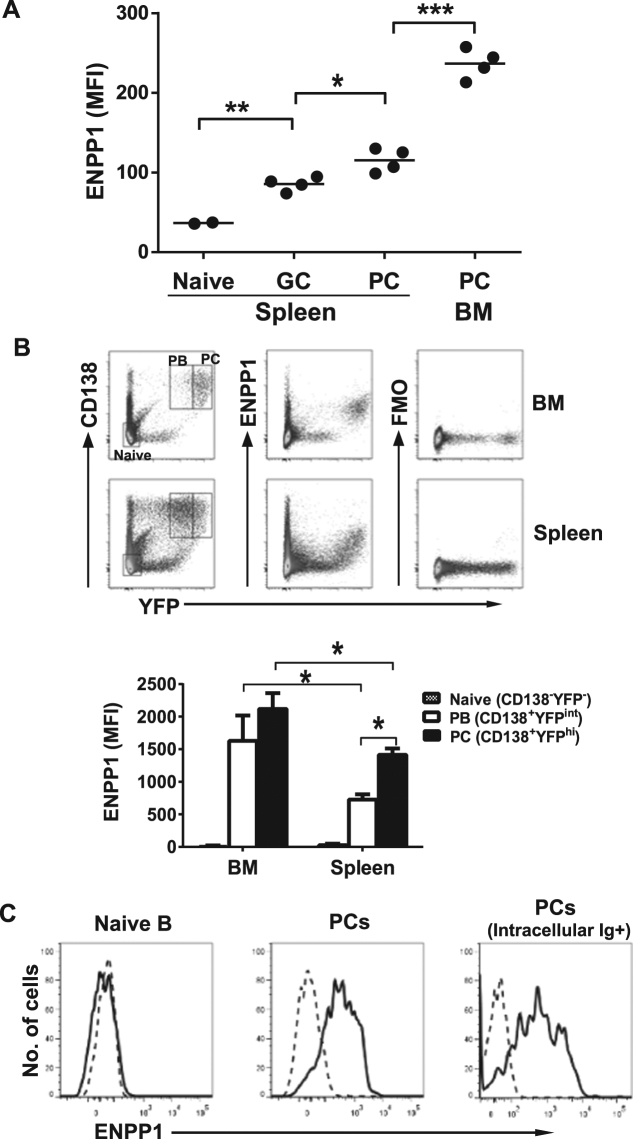
PCs express high levels of ENPP1. (**A**) Spleen and BM cells from B6 mice immunized with NP-KLH/alum for 2 wks were stained with antibodies to ENPP1, B220, GL7, CD138, and PNA and analyzed by flow cytometry. Cells were gated for naïve (B220^+^GL7^−^CD138^−^), GC (GL7^+^PNA^+^) and PC cells (B220^dull/−^CD138^+^). The expression levels of ENPP1 are depicted as mean fluorescence intensity (MFI). Each symbol represents a mouse. (**B**) Spleen and BM cells from Blimp1^Yfp/+^ mice immunized with NP-KLH for 2 wks were analyzed by flow cytometry. The top panel indicates the gating schemes used for defining naïve (CD138^−^YFP^−^), PBs (CD138^+^YFP^int^) and PCs (CD138^+^YFP^hi^). FMO, fluorescence minus one control for the anti-ENPP1 antibody. The bar graph (bottom) is the absolute MFI of ENPP1 of indicated cell subsets. Error bars are of 4 mice. (**C**) BM cells from a human adult female donor were pre-enriched for PCs with anti-human CD138 magnetic beads and stained and analyzed by FACS. Cells were gated for naïve (CD20^+^CD10^−^CD38^lo^CD19^+^IgM^+^), PCs (CD20^−^CD10^−^CD38^+^CD138^+^) and PCs (intracellular Ig) (CD20^−^CD38^+/hi^Intracellular Igκ/λ^+^), respectively. A non-paired two-tailed Student’s t-test was used. *p < 0.05, **p < 0.01, and ***p < 0.001.

Our previous analyses of ENPP1 expression on the surface of B lineage cells indicated that early and mature B cells express only low levels^37^. However, splenic GC B cells (GL7^+^PNA^+^) and PCs (B220^dull/-^CD138^hi^) exhibit markedly increased expression (^37^ and Fig. 1A). Interestingly, BM PCs expressed 2-fold more ENPP1 than their splenic counterparts (Fig. 1A). To confirm this finding, we analyzed Blimp1-YFP reporter mice (*Blimp1* ^*Yfp/+*^) in which plasmablasts (PBs) and mature PCs can be accurately distinguished by gating on B220^+^YFP^int^ and B220^dull^YFP^hi^ cells, respectively^38,39^. Fourteen days after immunization with 4-Hydroxy-3-nitrophenylacetyl hapten conjugated keyhole limpet hemocyanin (NP-KLH) and alum, splenic YFP^hi^ mature PCs expressed ~ 2-fold more ENPP1 than YFP^int^ PBs while there was nearly another 2-fold increase in ENPP1 expression by YFP^hi^ LLPCs in BM (Fig. 1B). Staining of human BM cells with a human-specific monoclonal anti-ENPP1 antibody^40^ also revealed high expression of ENPP1 on PCs compared with naïve B cells (Fig. 1C). These results thus demonstrated that high expression of ENPP1 is associated with LLPCs in BMs of both mice and humans.

### ENPP1 is dispensable for development of B and T cells

The high expression of ENPP1 in LLPCs prompted us to examine the function of ENPP1 in PC development by studying ENPP1-deficient mice (*Enpp1* ^*−/−*^). While *Enpp1* ^*−/−*^ mice have been extensively studied for skeletal, muscular and metabolic abnormalities^27,28,35,41–44^, we are unaware of studies focused on the immune system. First, we characterized the phenotypes and distributions of B and T cells in *Enpp1* ^*−/−*^ mice by flow cytometry. We found that the development of B and T cells was grossly normal in *Enpp1* ^*−/−*^ mice compared with *Enpp1* ^+/+^ littermates (designated wild-type or WT) (Figure S1A–C). Although the frequencies of BM pre-B and immature B cells were substantially higher in *Enpp1* ^*−/−*^ mice than in WT controls, the frequencies and absolute numbers of B cell subsets in the periphery were comparable between *Enpp1* ^*−/−*^ and WT mice (Figure S1). The mechanisms underlying the increased frequency of pre-B cells in ENPP1-deficient mice are currently unclear and warrant further investigation. Nevertheless, we conclude that ENPP1 is dispensable for B and T cell development in mice.

We next examined B cell proliferative responses to TLR ligands, including LPS and CpG oligodeoxynucleotides, or BCR ligation *in vitro*. Both *Enpp1* ^*−/−*^ and WT B cells proliferated to comparable extents following stimulation (Figure S2A). Finally, we examined T-independent (TI) immune response by immunizing mice with NP-LPS and NP-Ficoll. TI antigen responses are characterized by fast generation of SLPCs with transient production of low affinity antibodies. Both *Enpp1* ^*−/−*^ and WT mice generated equivalent antibody responses as assessed by NP-specific antibody levels in blood (Figure S2B and C). We therefore conclude that ENPP1 is dispensable for T-independent immune responses.

### ENPP1 deficiency affects development of LLPCs in BM following T-dependent immune responses

**Figure 2:**
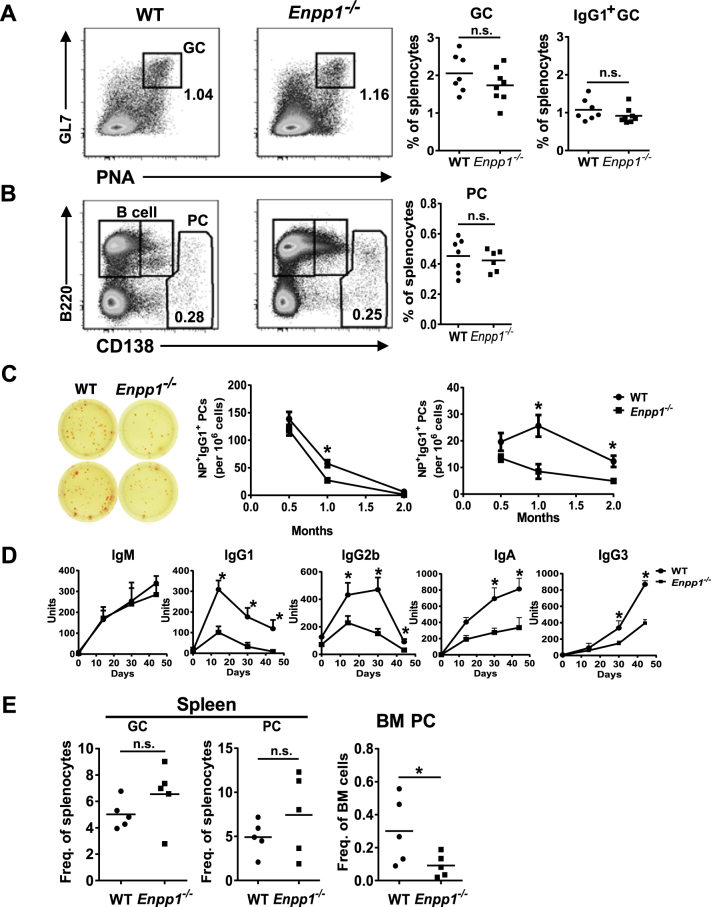
Enpp1^*−/−*^ mice have normal GC responses but impaired antibody production following immunization with TD antigens. (**A**,**B**) WT and Enpp1^*−/−*^ mice were immunized with NP-KLH/alum and were analyzed at 2 week by flow cytometry. The numbers are percentages of cells falling in each gate (dot plot histograms). Each symbol represents a mouse (right panels). (**C**) ELISPOT analysis of immunized mice as in (**A**) were performed at 0.5, 1 and 2 month following immunization. NP-specific IgG1^+^ PCs are shown. Error bars are of 6 mice. *p < 0.05. (**D**) The serum levels of NP-specific antibodies were measured by ELISA. Error bars are of 5–9 mice. *p < 0.05. (**E**) The mice indicated were infected with C. chabaudi for 8 days. Splenic and BM cells were stained and analyzed by flow cytometry. Each symbol represents a mouse. A non-paired two-tailed Student’s t-test was used. *p < 0.05. n.s., not significant.

We next examined T-dependent antigen responses in *Enpp1* ^*−/−*^ and WT mice by using a standard NP-KLH/alum immunization protocol for 2 wk. We first analyzed development of GCs and PCs by flow cytometry. The frequencies of GC B cells (GL7^+^PNA^+^) and switched IgG1^+^ GC B cells in spleens of WT and *Enpp1* ^*−/−*^ mice were comparable (Fig. 2A). The frequencies of splenic B220^dull/-^CD138^hi^ PCs were also comparable between *Enpp1* ^*−/−*^ and WT mice (Fig. 2B). Next, we assessed dynamic generation of NP-specific splenic and BM PCs by sensitive ELISPOT assays over a course of 2 mo following immunization. As shown in Fig. 2C, the frequencies of NP-specific IgG1-secreting PCs were significantly lower in spleens of *Enpp1* ^*−/−*^ mice than WT controls at 1 mo. At 2-mo, splenic PCs numbers had declined to base line levels for both groups of mice (Fig. 2C), consistent with the view that splenic PCs were primarily SLPCs. Interestingly, the frequencies of NP-specific IgG1^+^ PCs in the BM were significantly lower in *Enpp1* ^*−/−*^ mice than in WT controls at both the 1- and 2-mo time points, perhaps initiating as early as 2-wk after immunization (Fig. 2C). Consistent with the changes in PC numbers, the levels of class-switched serum NP-specific antibodies were also lower in *Enpp1* ^*−/−*^ mice than in WT controls (Fig. 2D). Taken together, these results indicate that during a T-dependent immune response, while the development of GCs was normal in *Enpp1* ^*−/−*^ mice, the continuing production and/or persistence of BM PCs was impaired in the absence of ENPP1.

To rule out the possibility that the compromised generation of BM LLPCs in *Enpp1* ^*−/−*^ mice was restricted to responses to a single T-dependent antigen, we employed a malaria parasite infection model. Previous studies demonstrated that infection of mice with plasmodium *Chabaudi (C*.*) chabaudi* induced rapid and robust immune responses with high levels of PCs in spleen and BM^45,46^. Consistent with these reports, we observed that mice infected with *C*. *chabaudi* for 8 d produced ~10 times more splenic PCs than those immunized with NP-KLH (Fig. 2E vs. Figure 2B). Whereas the magnitude of inflammation (spleen weights) and the frequencies of splenic GCs, PBs, PCs and blood PCs were comparable between infected WT and *Enpp1* ^*−/−*^ mice (Fig. 2E and Figure S3), the frequencies of BM PCs were significantly lower in *Enpp1* ^*−/−*^ mice than in WT controls (Fig. 2E).

### The effect of ENPP1 on BM PC development is B cell-intrinsic

**Figure 3:**
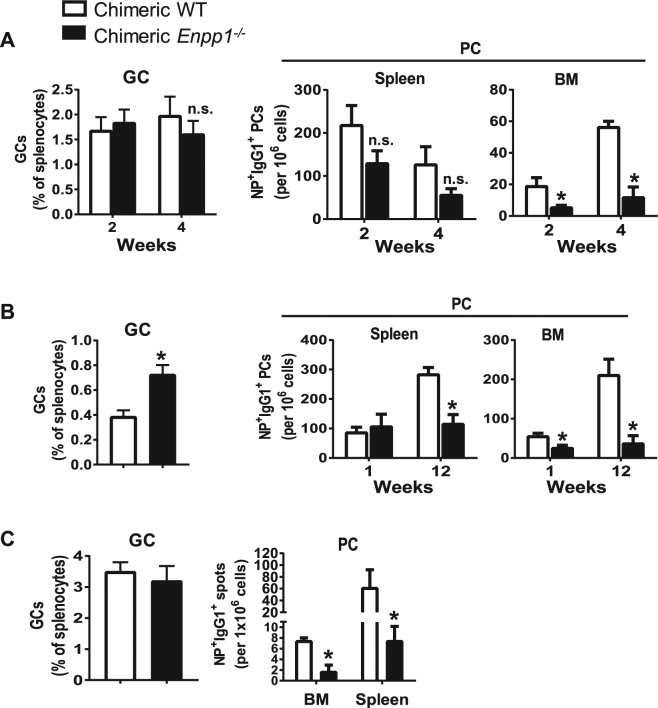
Decreased persistence of PCs in Enpp1^*−/−*^ chimera mice. Chimeric mice were generated by reconstituting lethally irradiated CD45.1 (**A** and **B**) or Rag1^*−/−*^ (**C**) mice with BM HSCs sort-purified from WT or Enpp1^*−/−*^ mice. The HSC reconstituted mice were immunized with NP-KLH and alum once (**A** and **C**) or twice (B). (**A**–**C**) GC B cells were analyzed by flow cytometry as depicted in Fig. 2A. (A–C) NP-specific PCs in BM and spleen were enumerated by ELISPOT. Error bars are 3–7 mice per group. Data are representative of 2–4 independent experiments. A non-paired two-tailed Student’s t-test was used. *p < 0.05. n.s., not significant.

To determine whether the impaired generation of BM PCs in *Enpp1* ^*−/−*^ mice was due to cell-intrinsic or -extrinsic mechanisms, we generated chimera mice by reconstituting lethally irradiated CD45.1 B6 mice with BM HSCs (sorted as lineage^-^cKit^+^Sca-1^+^IL7R^−^) as we reported previously^47^. Eight weeks after transfer, most (>90%) splenocytes of the recipients exhibited the donor origin as assessed by CD45.1 staining. We immunized these mice with NP-KLH and alum and assessed GC responses by flow cytometry and ELISPOT assays. The frequencies of GCs were indistinguishable between recipients of WT HSCs (designated chimeric WT) and *Enpp1* ^*−/−*^ HSCs (designated chimeric *Enpp1* ^*−/−*^) at 2 and 4 week following immunization (Fig. 3A), consistent with the results of immunized conventional mice (Fig. 2A). However, the frequencies of NP-specific PCs were significantly lower in the BM of chimeric *Enpp1* ^*−/−*^ mice than chimeric WT controls. In spleens of recipient mice, the frequencies of NP-specific PCs also tended to be lower in chimeric *Enpp1* ^*−/−*^ mice than WT controls (Fig. 3A). Interestingly, at 1 wk following a boost immunization, chimeric *Enpp1* ^*−/−*^ mice generated more GCs than WT controls. In this case, the frequencies of splenic PCs were slightly increased but the numbers of BM PCs were significantly decreased in chimeric *Enpp1* ^*−/−*^ mice compared with chimeric WT controls. At 12 wk following boost immunization, the frequencies of PCs were significantly lower in both spleen and BM of chimeric *Enpp1* ^*−/−*^ mice than chimeric WT controls (Fig. 3B). These data indicate that long-term survival of PCs was impaired in *Enpp1* ^*−/−*^ mice regardless of the location.

It has been reported that LLPCs are radiation resistant^6^ and that newly generated PCs need to expel pre-existing PCs before they can establish their own survival niches^48^. Therefore, it was possible that the immunization-induced ENPP1-deficient PCs were defective in displacing pre-existing, radiation-resistant PCs in CD45.1 B6 recipients (Fig. 3A and B). To examine this possibility, we generated chimeric mice using *Rag1* ^*−/−*^ mice devoid of pre-existing BM PCs as recipients. The mice were examined 1 mo following immunization with NP-KLH and alum. In this case, similar to above results (Fig. 3A,B), chimeric *Enpp1* ^*−/−*^ mice generated normal numbers of GCs but significantly lower numbers of PCs in both BM and spleen compared with controls (Fig. 3C). Taken together, these results support a B cell-intrinsic effect of ENPP1 on PC development and survival.

### ENPP1 deficiency does not affect migration of PBs ***in vivo*** and ***in vitro***

**Figure 4:**
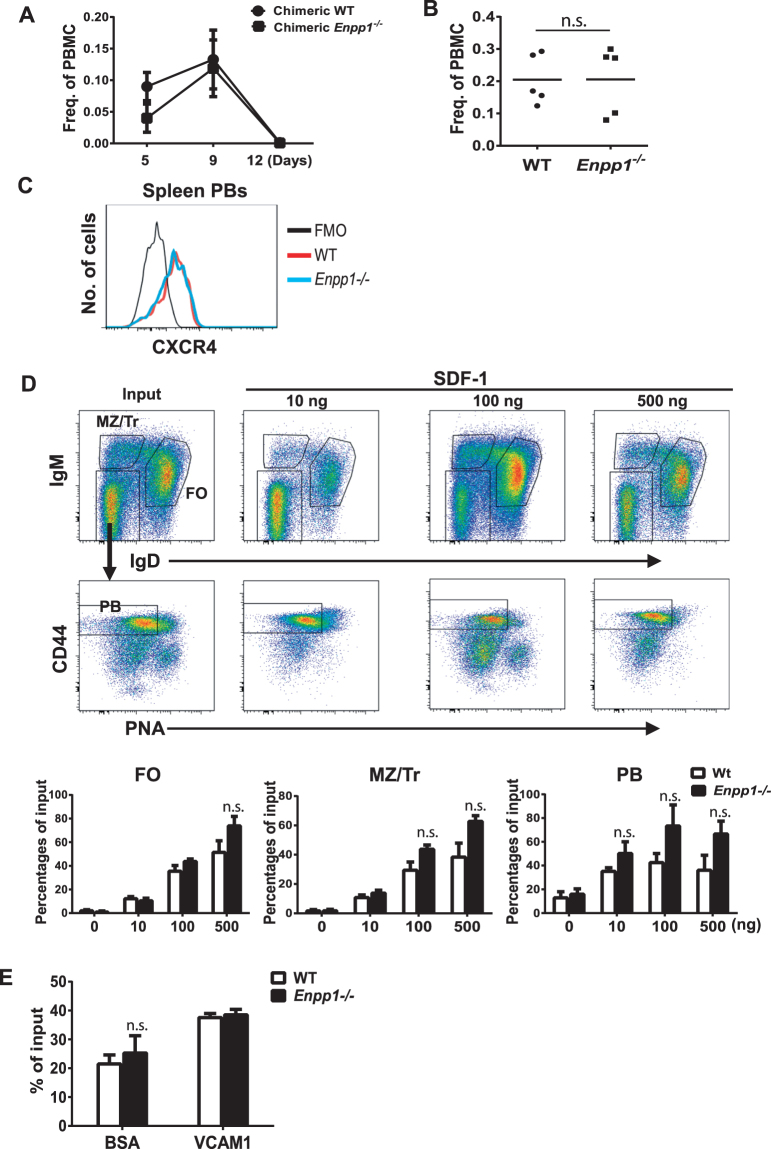
ENPP1 deficiency does not affect migration of PBs. (**A**) Normal generation of PBs in Enpp1^*−/−*^ chimera mice. Chimeric mice as in Fig. 3A were immunized with NP-KLH and alum. PBs (B220^+^IgM^lo/−^IgD^−^CD44^+^PNA^lo^NP^+^) in blood at the indicated times following immunization were analyzed by flow cytometry. Error bars are of 5–10 mice per group. (**B**) PBs in blood of *C. chabaudi* infected mice as in Fig. 2E were analyzed by flow cytometry. Each symbol represents a mouse. (**C**) Expression of CXCR4 on splenic PBs were analyzed by flow cytometry. Representative data of 3 independent experiments are shown. (**D**) Splenic B cells from mice immunized with NP-KLH for 7 days were purified by negative selection and subjected to transwell assays in the presence of different concentrations of SDF-1. The cells migrating to the lower chamber were stained and analyzed by FACS. Top panel, gating strategy for defining the indicated B cell subsets. Lower panel, cell counts of triplicate assays. (**E**) A similar transwell assay as in (**D**) was performed with transwells that were pre-coated with VCAM1 or BSA. Error bars are of 3 mice. Data are representative of two independent experiments (**D** and **E**). A non-paired two-tailed Student’s t-test was used. n.s., not significant.

To determine whether the reduced frequency of BM PCs in *Enpp1* ^*−/−*^ mice was due to an inability of ENPP1-deficient PBs to migrate into the BM, we first measured the frequencies of circulating PBs in mice immunized with NP-KLH or plasmodium-infected mice. PBs were detected by flow cytometry as B220^+^IgM^−^IgD^−^CD44^+^PNA^lo^NP^+^ as previously reported^49^ (also refer to Fig. 4D). As shown in Fig. 4A, the frequencies of blood PBs peaked 9 d after immunization. There was no significant difference in the frequencies of blood PBs between chimeric *Enpp1* ^*−/−*^ and WT mice (Fig. 4A). The frequencies of blood PBs in *Enpp1* ^*−/−*^ and WT mice infected with *C*. *chabaudi* were also equivalent (Fig. 4B). Therefore, these data suggest that ENPP1 deficiency did not affect the output of PBs in peripheral blood of mice under both immunization and infection conditions.

Homing of circulating PBs to the BM is mediated by well-characterized biological processes involving vascular adhesion and extravasation guided by CXCR4-CXCL12-mediated chemotaxis. Figure 4C shows that the levels of CXCR4 expressed by splenic PBs were equivalent between *Enpp1* ^*−/−*^ and WT mice. Transwell migration assays revealed that comparable numbers of ENPP1-deficient and -sufficient B cells migrated toward SDF-1 (CXCL12) in a dose-dependent fashion (Fig. 4D). Pre-coating the transmembrane with VCAM1 did not affect transwell migration either (Fig. 4E). Finally, we analyzed expression of 34 genes involved in leukocyte trafficking in BM PCs by qPCR and flow cytometry. There were no significant differences in expression levels of these genes between WT and ENPP1-deficient PCs (Figure S4 and data not shown).

Finally, we measured the frequencies of Ki67^+^ BM PCs in WT and *Enpp1* ^*−/−*^ mice immunized with NP-KLH/alum for 10 days. Ki67 is a nuclear protein associated with recent cell division and is expressed in newly formed PCs in the BM^50^. While the frequencies of Ki67^+^ PCs were equivalent in the spleens of WT and *Enpp1* ^*−/−*^ mice, the frequencies of Ki67^+^ PCs were proportionally slightly increased in the BM of *Enpp1* ^*−/−*^ mice compared with WT controls (Fig. S5). Taken together, these data suggest that ENPP1-deficient and –sufficient PBs and PCs are indistinguishable in homing capacity.

### ENPP1-deficiency impairs glucose-mediated metabolic activity of PCs

**Figure 5:**
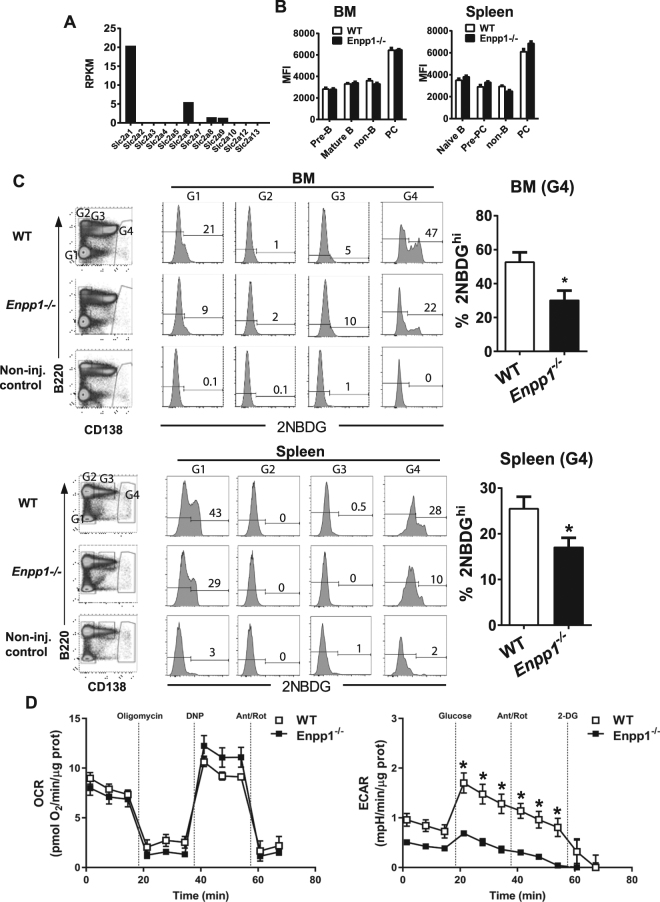
Impaired glucose uptake and metabolism in PCs of Enpp1^*−/−*^ mice. (**A**) Expression levels of glucose transporters in a mouse PCT cell line were analyzed by RNA-seq. RPKM, reads per kilobase of transcript per million mapped reads. (**B**) Expression levels of GLUT1 in B cell subsets of BM and spleen were measured by flow cytometry. MFI, mean fluorescence intensity. Error bars are of 3 mice in 2 independent experiments. (**C**) 2NBDG uptake *in vivo* by the indicated cell populations was quantitated by flow cytometry. The numbers are percentages of cells falling in each gate. Error bars of 4 mice per group. Data are representative of 2 independent experiments. A non-paired two-tailed Student’s t-test was used. *p < 0.05. (**D**) ENPP1-deficient PCs have reduced levels of glycolysis than wild-type PCs. Mitochondrial stress test to measure oxygen consumption rate (OCR, left panel) and glycolysis stress test to measure extracellular acidification rate (ECAR, right panel) of freshly isolated PCs from mice immunized with NP-KLH for 9 days. Error bars are technical triplicates. A non-paired two-tailed Student’s t-test was used. *p < 0.01. Data are representative of 2 independent experiments with similar results.

Recently, Lam *et al*. reported that LLPCs take up more glucose than SLPCs and are more dependent on glucose for glycolysis and survival than SLPCs^17^. To determine whether ENPP1 deficiency would affect glucose uptake in PCs, we first examined the expression levels of glucose transporters in PCs. Among the 12-member glucose transporter (GLUT) family, GLUT1 was the dominant transporter expressed by PCs as determined by RNAseq analyses of an ENPP1-deficient plasmacytoma cell line (Fig. 5A). The high expression of GLUT1 in PCs was confirmed at the protein level by flow cytometric studies of BM and splenic PCs stained with an anti-GLUT1 antibody (Fig. 5B). Regardless of the location, both BM and splenic PCs expressed 2-fold higher levels of GLUT1 than other B and non-B cell subsets, suggesting a marked surge in demand for glucose for PC function. We next measured glucose uptake by PCs *in vivo* by injecting mice with 2NBDG, a fluorescent glucose analog, followed by flow cytometric analyses. Compared with WT controls, ENPP1-deficient mice exhibited significantly lower frequencies of 2NBDG^+^ PCs in both the BM and spleen (Fig. 5C). Consistent with a previous report^17^, BM PCs contained twice as many 2NBDG^bri^ cells than splenic PCs (Fig. 5C). Because there was only about 25% of splenic PCs labeled with 2NBDG at a high level, this data is consistent with the view that the majority of splenic PCs are short-lived. In summary, our data indicate that the fewer 2NBDG^bri^ PCs in *Enpp1* ^*−/−*^ mice parallel results showing that ENPP1-deficent mice generated fewer LLPCs (Fig. 3).

To further understand the role of ENPP1 in the regulation of metabolic activity essential for PC survival, we performed mitochondrial stress test to determine oxidative phosphorylation (measured as oxygen consumption rate (OCR)) and glycolysis stress test (measured as extracellular acidification rate (ECAR)) in purified PCs from immunized WT and *Enpp1* ^*−/−*^ mice. For the mitochondrial stress test, cells were treated sequentially with oligomycin (an inhibitor of the ATP synthase), 2,4-Dinitrophenol (DNP, an uncoupler) and rotenone plus antimycin A (inhibitor of complex I and III of the respiratory chain respectively). For the glycolysis stress test, glucose-starved cells were treated sequentially with glucose, rotenone plus antimycin A and 2-deoxy-D-glucose (2-DG, an inhibitor of glycolysis)^51^. As shown in Fig. 5D, splenic PCs of *Enpp1* ^*−/−*^ mice exhibited strongly reduced ECAR compared with WT controls even though *Enpp1* ^*−/−*^ PCs displayed a modest but non-significant increase in maximal oxygen consumption after DNP treatment (Fig. 5D). LPS-stimulated B cells, which were mostly proliferating PBs, also exhibited similar patterns of metabolic changes with moderate reductions of ECAR in ENPP1-deficient cells (Figure S6). Together these results suggest that impaired glucose uptake in ENPP1-deficient PCs leads to a defect in glycolysis without a compensatory increase in either basal or maximal respiratory capacity. This loss of glycolytic capacity and no significant change in oxidative phosphorylation capacity likely renders ENPP1-deficient PCs at a great survival disadvantage *in vivo*.

## Discussion

The development of durable protective antibody responses to vaccines or infections is critically dependent on the generation of LLPCs in the BM. Although remarkable progress has been made in defining the environmental niches, cytokines, chemokines and transcriptional programs that characterize these cells (reviewed in^52^) it is only recently that metabolic factors conferring survival to LLPCs have been explored. Lam *et al*. (27) elegantly demonstrated that LLPCs are distinguished from SLPCs by their increased capacity to import and utilize glucose as a source of mitochondrial respiration, thereby promoting survival and high-level Ig secretion. Unanswered, however, was the question of how these unique attributes of LLPC are regulated.

Here we identify ENPP1 as a crucial regulator promoting survival of LLPC in the BM. While the frequencies of PCs in the spleens of ENPP1-deficient mice were only moderately reduced compared with ENPP1-sufficient controls in the early phase of a TD immune response, a significant reduction of LLPCs was found in the BM of *Enpp1* ^*−/−*^ mice. This defect in BM LLPC survival/persistence was PC intrinsic as assessed by studying BM chimeric mice. Similar to the results of conventional mice, chimeric mice also produced comparable levels of GC B cells following immunization with NP-KLH. However, the frequencies of long-lived antigen-specific BM PCs were significantly lower in chimeric *Enpp1* ^*−/−*^ mice than WT controls. Importantly, we found that PCs that reached the BM of *Enpp1* ^*−/−*^ mice took up less glucose than BM PCs of WT controls. Considering the fact that BM PCs express two-fold more ENPP1 than splenic PCs (Fig. 1), we reasoned that ENPP1 allows BM LLPCs to consume more glucose to better facilitate higher amount of antibody production and longer time of survival. This view is consistent with the finding that LLPCs rely more heavily on glucose for glycolysis and antibody production than splenic SLPCs^17^. Indeed, ENPP1-deficient PCs exhibited greater reduction in ECAR (Fig. 5), a parameter indicating an impaired glycolysis pathway, which would be expected to lead to reduced levels of energy production thereby poor survival of PCs *in vivo*.

One important question related to the decreased numbers of BM PCs in *Enpp1* ^*−/−*^ mice is whether the ENPP1-deficient PBs are capable of homing to the BM in the first place. Our analyses indicated that the frequencies of PBs in peripheral blood of *Enpp1* ^*−/−*^ mice were within the normal range following immunization with NP-KLH or infection with *C*. *chabaudi*. Homing of PBs to the BM is mediated by the chemokine CXCL12 and the receptor CXCR4 on PC^53^ as well as adhesion molecules including integrins^54^. Our analyses indicated that expression of CXCR4 on PBs and PCs was comparable between *Enpp1* ^*−/−*^ and WT mice. The chemotactic response of PBs to CXCL12 in *vitro* was also comparable between these mice. In addition, we analyzed 34 adhesion molecules, including a panel of 10 integrin molecules known to be important for lymphocyte trafficking, and found no differences in expression between PCs of *Enpp1* ^*−/−*^ and WT mice. The expression levels of other adhesion molecules including VCAM1, CD44 and CD144 were also normal on PCs of *Enpp1* ^*−/−*^ mice as detected by flow cytometry (data not shown). Considering the results that ENPP1-sufficient and -deficient B cells including PBs were indistinguishable in transwell migration assays (Fig. 4), it is unlikely that the homing of ENPP1-deficient PBs to the BM was impaired.

The functions of ENPP1 have been broadly studied in the fields of bone formation and type 2 diabetes. Previous studies revealed that osteoblasts and chondrocytes express high levels of ENPP1^20,24,55^. ENPP1 plays a negative regulatory role in bone mineralization mediated by PPi, a product from ATP hydrolysis mediated by ENPP1^56^. While direct evidence is still lacking, it is possible that high expression of ENPP1 on PCs may suppress new bone formation to preserve survival niches. In addition to the catalytic activity of ENPP1, ENPP1 has been found to modulate insulin receptor activity and glucose homeostasis (reviewed in^57^). By directly interacting with the alpha chain of the insulin receptor, ENPP1 downregulates the insulin signaling pathway causing insulin resistance^58,59^. Increased ENPP1 expression in the liver of ENPP1 transgenic mice correlates with insulin resistance and glucose intolerance^60^. These data suggest that ENPP1 is critical for insulin-stimulated glucose metabolism. While the responsiveness of PCs to insulin stimulation remains unclear, our findings that ENPP1-deficient PCs fail to gain the LLPC phenotype by consuming more glucose for antibody production and survival highlight the possibility that ENPP1 is a critical regulator for glucose-dependent metabolic processes in PCs.

The dominant substrate of ENPP1 is ATP. ATP is released from cells undergoing stress, inflammatory stimulation, hypoxia or death^61^. Extracellular ATP is an important mediator of inflammation with broad effects on cellular functions^62^. Given that the BM microenvironment is enriched for developing cells with a high frequency of apoptotic events, rapid degradation of extracellular ATP would be expected to minimize the negative effect of ATP on LLPCs. Consistent with this view, preliminary experiments suggest that ENPP1 suppresses exogenous ATP-induced oxygen consumption but enhances glycolysis in LPS-induced PBs (data not shown). In addition, hydrolysis of ATP by ENPP1 also produces PPi, a high-energy substitute for ATP that can be transported freely crossing cell membranes. PPi has been found to be beneficial for sperm survival and to increase *in vitro* fertilization rates^63^. Therefore, the beneficial effect of ENPP1 on PC survival could be multifaceted.

Controlling plasma cell-related diseases such as multiple myeloma has been very difficult, due in part to a paucity of good target molecules on the cell surface. Our findings suggest that ENPP1 could be an attractive therapeutic target for intervention in PC-related diseases if PC specificity could be achieved. In addition, in view of the importance of LLPC for enduring antibody-mediated immunity to vaccines and infections, further understandings of the signals promoting PC longevity could prove to be informative for rational vaccine design.

## Materials and Methods

### Mice and cells

C57BL/6 J (B6) (CD45.2 and CD45.1) and *Rag1* ^*−/−*^ mice were purchased from the Jackson Laboratory (Bar Harbor, ME). *Enpp1* ^*−/−*^ mice were described previously^27^. Blimp-1 YFP reporter mice were obtained from Dr. Eric Meffre at Yale University. All mice were housed under pathogen-free conditions. All animal studies were performed under protocols of LIG-16 approved by the NIAID IACUC and all experiments were done in accordance with the NIH guidelines and regulations.

To generate chimeric mice, CD45.1 and *Rag1* ^*−/−*^ mice were irradiated at 1,100 (with a short interval) and 500 rad, respectively, one day before i.v. injection with 0.6–2 × 10^4^ sort-purified HSCs (Lin^-^Sca1^+^c-Kit^+^IL7R^−^) from WT or *Enpp1* ^*−/−*^ mice, as previously reported^47^. The efficiency of reconstitution was verified by FACS analyses of blood for frequencies of B and T cells shortly before mice were immunized 8 wks later.

Human bone marrow cells were purchased from Allcells (Alameda, CA) and processed for antibody staining and flow cytometry analysis as described below.

### Immunization and ELISA

Mice were immunized i.p. with NP-LPS (50 μg), NP-Ficoll (20 μg), NP_23_-KLH (50 μg in alum) (Biosearch Technologies, Novato, CA). At different time points after immunization, the mice were bled and the serum antibodies were measured by ELISA. Briefly, pre-coated plates with NP_26_-BSA (Biosearch Technology) were incubated with diluted serum samples and developed with HRP-conjugated mouse isotype-specific Ab (Southern Biotech) and substrate o-phenylenediamine dihydrochloride (Sigma-Aldrich). The plates were read at 450 nm using an ELISA plate reader.

### Flow cytometry (FACS)

Single cell suspensions from thymus, BM, spleen and peritoneum of mice were prepared and stained with fluorochrome-labeled mAbs using standard procedures. A cell viability dye, 7AAD or Fixable Violet dye (Invitrogen), was routinely included. All Abs, except as indicated, were purchased from BD-Biosciences (San Diego, CA). The anti-human ENPP1 (clone 2D7, provided by Dr. James Goding, Monash University, Victoria, Australia) and mouse ENPP1 (clone YE1/19.1, provided by Dr. Fumio Takei, Terry Fox Laboratory, Canada) were labeled with allophycocyanin (APC)^37^. Cells were analyzed using a FACSCalibur, LSR II (BD Biosciences) or sorted on a FACS Aria sorter (BD Biosciences).

### Quantitative Real-time RT-PCR (qPCR)

Total RNA from sorted follicular (FO) (B220^+^CD23^+^CD21^int^), GC (PNA^+^GL-7^+^) and PCs (B220^dull/-^CD138^bri^) was reverse-transcribed with SuperScript^TM^ II enzymes (Invitrogen, Carlsbad, CA). The cDNA was subjected to real-time PCR analysis using an ABI Prism 7900HT system and the SYBR Green PCR Master Mix reagents (Applied Biosystems). PCR primer sequences were: *Enpp1* forward, 5′-TTAATGTGTGACTTACTGGGTTTGATC-3′; *Enpp1* reverse, 5′-GGTTGAGGCTGCCATGACTT-3′. All other primers were described previously^64^.

### ***In vitro*** treatment

Splenic B cells were purified by negative selection using magnetic-activated Dynal beads (Invitrogen, Carlsbad, CA) and labeled with CFSE using a standard protocol. Cells (1–2 × 10^6^/ml) were cultured in 24-well plates for different periods of time with 20 μg/ml of LPS (Sigma-Aldrich), 1 μg/ml of CpG ODN 1826 (Invivogene, San Diego, CA), or 10 μg/ml of F(ab’)_2_ anti-μ Ab (Jackson ImmunoResearch Laboratory) plus 1 μg/ml of anti-CD40 Ab (Southern Biotech). The cells were stained with 7AAD and annexin V and analyzed by FACS.

### ELISPOT assays

Spleen and BM PCs were quantified by NP-specific Ig ELISPOT assays. Briefly, aliquots of 1.25–5.0 × 10^5^ spleen and BM cells were plated in triplicate in NP_26_-BSA pre-coated 96-well PVDF membrane plates (Millipore) and were incubated overnight at 37 °C in 5% CO_2_. The plates were washed with PBS containing 0.05% Tween-20 and incubated with HRP-conjugated anti-mouse IgM or IgG1 (Jackson ImmunoResearch Laboratory), followed by reaction with FAST 5-bromo-4-chlor-3-indolyl phosphate/NBT chromogen substrate (Sigma-Aldrich). The plates were scanned with a CTL-ImmunoSpot^®^ S5 Core Analyzer (Cellular Technology) and analyzed by ImmunoSpot^®^ Software 4.0 (Cellular Technology).

### Glucose uptake and Metabolic analyses

For *in vivo* 2NBDG labeling, mice were injected i.v. with 100 μg 2NBDG (Cayman Chemicals) in PBS and euthanized 15 minutes later. BM and splenic cells were isolated, stained and analyzed by flow cytometry.

For extracellular flux assays, we used a standardized protocol to measure OCR and ECAR by a Seahorse XF 96 analyzer described in great detail recently^51^. *Ex vivo* PCs were purified from 22 mice immunized with NP-KLH and alum for 9 days using a CD138^+^ plasma cell isolation kit (Miltenyi Biotech). 8 × 10^5^ cells were plated in 96-well Cell-Tack (Corning)-coated Seahorse plates and analyzed.

### Generation of ENPP1-deficient plasmacytoma

Pristane-induced plasmacytoma was generated in *Enpp1* ^*−/−*^ *Myc*/*Bclxl* transgenic (Tg) mice that were generated by several rounds of breeding of *Enpp1* ^*+/−*^ with Em-BCL_xL_ Tg^65^ and heterozygous C.iMyc^Cα^ ^66^ mice. Mice were injected with 0.4 ml of pristane and monitored for signs of ascites. Plasma cell tumors were diagnosed using Wright-Giemsa–stained cytofuge preparations of ascites. When mice carried more than 100 tumor cells per field, they were euthanized and peritoneal neoplastic tissues, ascites and spleens were collected, cell suspensions were prepared and cultured with complete RPMI 1640 medium in the presence of IL6 till cell lines were established. The phenotype of the cell lines was assessed by flow cytometry. One cell line (10659) used in this study was B220^−^, CD19^−^, CD138^+^, IgM^lo/−^, Igκ^lo^, IgD^−^, CD5^−^, CD23^−^, CD21^−^, integrin αV^+^, β1^+^, β3^+^, and CD11b^lo^.

### Statistical analysis

Data were analyzed using 2-tailed Student’s *t* test (2-tailed). *P* < 0.05 is regarded as statistically significant.

## Electronic supplementary material

Supplemental information
