# Supplementary material for: ATP-degrading ENPP1 is required for survival (or persistence) of long-lived plasma cells
Source: Sci Rep. 2017 Dec 19;7:17867. doi: 10.1038/s41598-017-18028-z (PMC5736562; doi:10.1038/s41598-017-18028-z)
Supplement: Supplementary file 1 — Supplemental information [file 41598_2017_18028_MOESM1_ESM.pdf]

Supplementary Information for:

ATP-degrading ENPP1 is required for survival (or persistence) of long-lived plasma cells

Hongsheng Wang[1], Ines Gonzalez-Garcia[1,2], Javier Traba[3], Shweta Jain[1], Solomon Conteh[4], Dong-Mi Shin[5], Chenfeng Qi[1], Yuanyuan Gao[1], Jiafang Sun[1], Sungyun Kang[1,8], Sadia Abbasi[1], Zohreh Naghashfar[1], Jeongheon Yoon[1,6], Wendy DuBois[7], Alexander L. Kovalchuk[1], Michael N. Sack[3], Patrick Duffy[4], and Herbert C. Morse III[1]

[1] Laboratory of Immunogenetics, National Institute of Allergy and Infectious Diseases, National Institutes of Health, Rockville, MD, USA 20852.

[2] Current address: Celgene Institute of Translational Research Europe, Parque Tecnológico Cartuja 93, c/ Isaac Newton n.4, E-41092 Seville, Spain.

[3] Laboratory of Mitochondrial Biology and Metabolism, National Heart, Lung and Blood Institute, National Institutes of Health, Bethesda, MD, USA 20814.

[4] Laboratory of Malaria Immunology and Vaccinology, National Institute of Allergy and Infectious Diseases, National Institutes of Health, Rockville, MD, USA 20852.

[5] Department of Food and Nutrition, Seoul National University, Seoul 151-742, Korea.

[6] Current address: Department of Medicine, Uniformed Services University of the Health Sciences, Bethesda, MD 20814.

[7]Laboratory of Cancer Biology and Genetics, National Cancer Institute, National Institutes of Health, Bethesda, MD, USA 20814.

[8] Current address: Department of Biology, Indiana University, Myers Hall 230, 915 E. 3rd St., Bloomington, IN 47405.

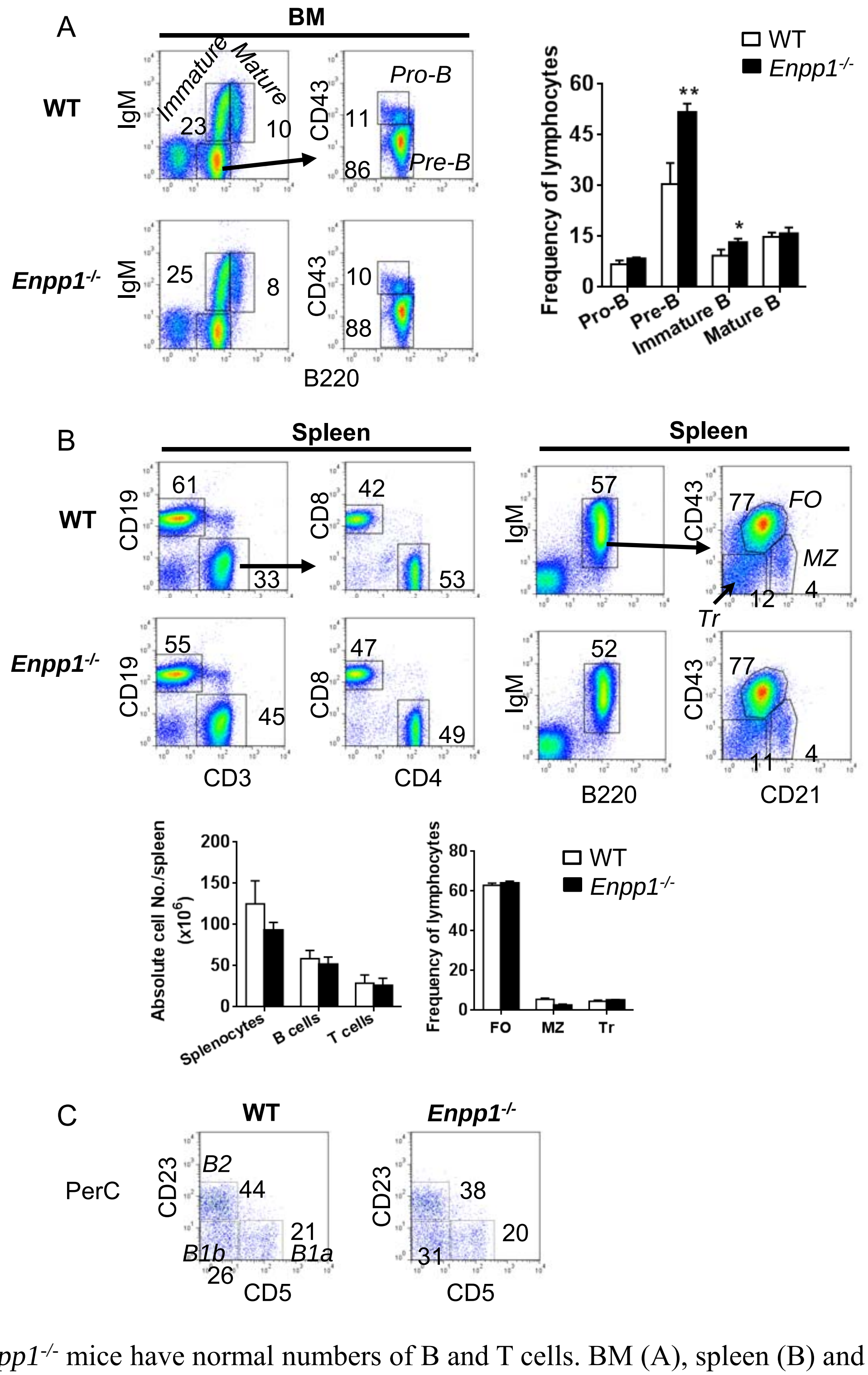

Figure S1. *Enpp1*$^{-/-}$ mice have normal numbers of B and T cells. BM (A), spleen (B) and peritoneal (PerC) cells (C) were stained with the indicated Abs and analyzed by FACS. The numbers are percentages of cells falling in each gate. MZ, marginal zone; FO, follicular; Tr, transitional. The bar charts are data of 5-7 mice per group. Data are representative of at least five independent experiments. *p<0.05, **p<0.01.

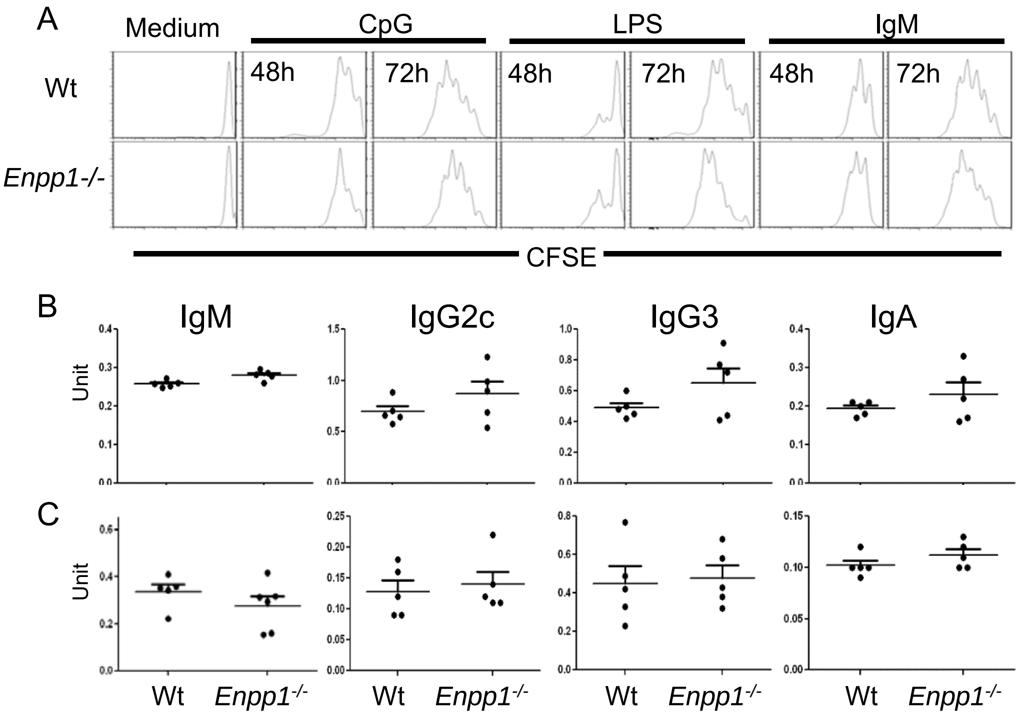

Figure S2. ENPP1-deficient mice exhibited normal T-independent immune responses. (A) Purified splenic B cells from WT and *Enpp1*$^{-/-}$ mice were labeled with CFSE and stimulated with LPS (20 μg/ml), CpG (1 μg/ml) or F(ab')$_2$ anti-IgM Ab (10 μg/ml) for 48 or 72 hrs. The cells were then stained with 7AAD and analyzed by FACS. Data are representative of 3 independent experiments. (B and C) Mice were immunized with 50 μg of NP-LPS (B) or 20 μg of NP-Ficoll (C). Sera were collected one week after immunization. The presence of NP-specific antibodies was quantified by ELISA. The values are arbitrary units calculated from a standard curve. Each symbol represents a mouse.

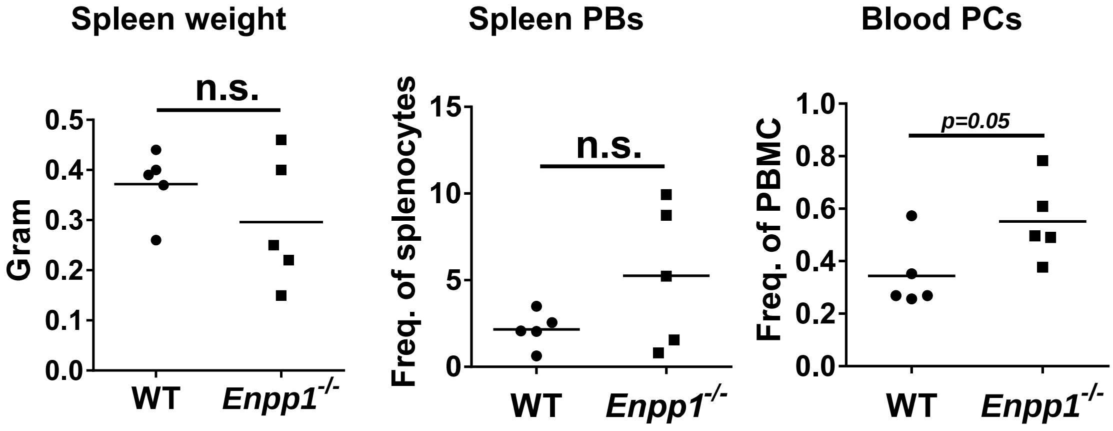

Figure S3. Inflammatory response to *C. chabaudi* infection. Mice were infected with the parasite for 8 days. Blood and spleen cells were analyzed by flow cytometry to quantify PBs and PCs. Each symbol represents a mouse.

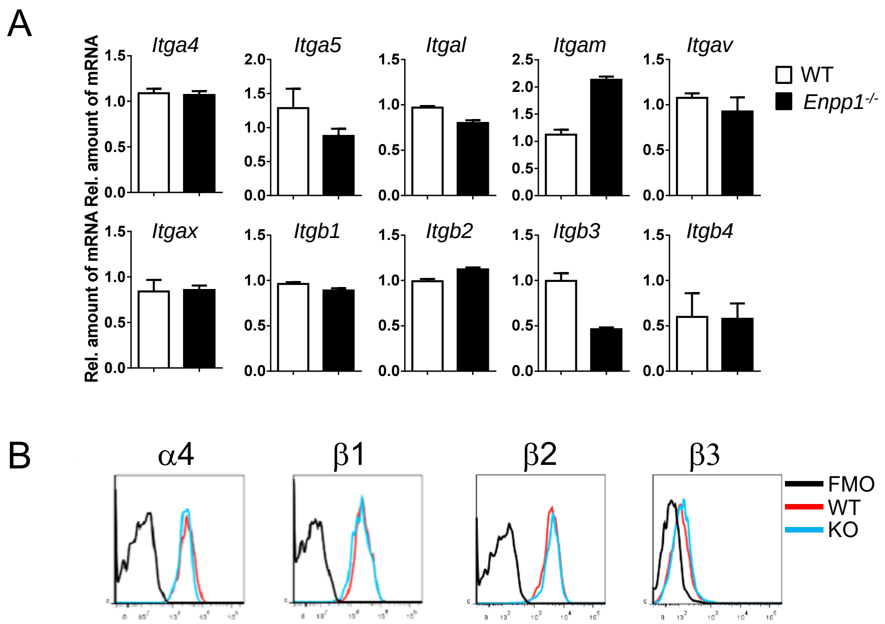

Fig. S4. Expression levels of integrin family members in PCs of WT and *Enpp1*$^{-/-}$ mice. (A) BM PCs were sort-purified and the expression levels of indicated integrin were measured by qPCR. Error bars are technical triplicates. (B) The expression levels of α4, β1, β2 and β3 integrin in BM PCs were analyzed by flow cytometry. Cells were gated on B220$^{lo/-}$CD138^{+} PCs. Data represent two independent experiments.

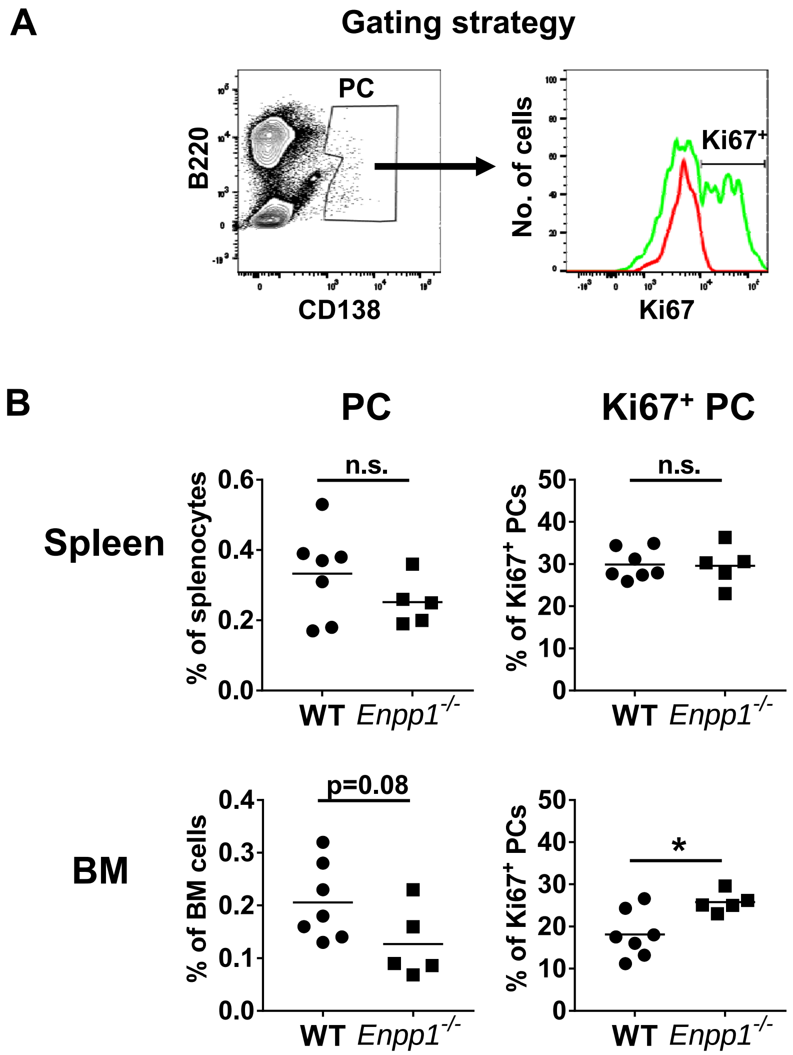

Fig. S5. Intracellular Ki67 expression in PCs. Mice were immunized with NP-KLH/alum for 10 days. BM and splenocytes were stained with antibodies against B220 and CD138, followed by fixation, permeabilization, and staining with anti-Ki67-Alexa647. The cells were analyzed by flow cytometry. (A) Gating strategy used to identify Ki67^{+} cells. All cells were gated on viable singlets. Red line is background. (B) Number of cells in each gate from spleen (top) and BM (bottom). Each symbol represents a mouse. A two-tailed t-test was used. *p<0.05. n.s., not significant.

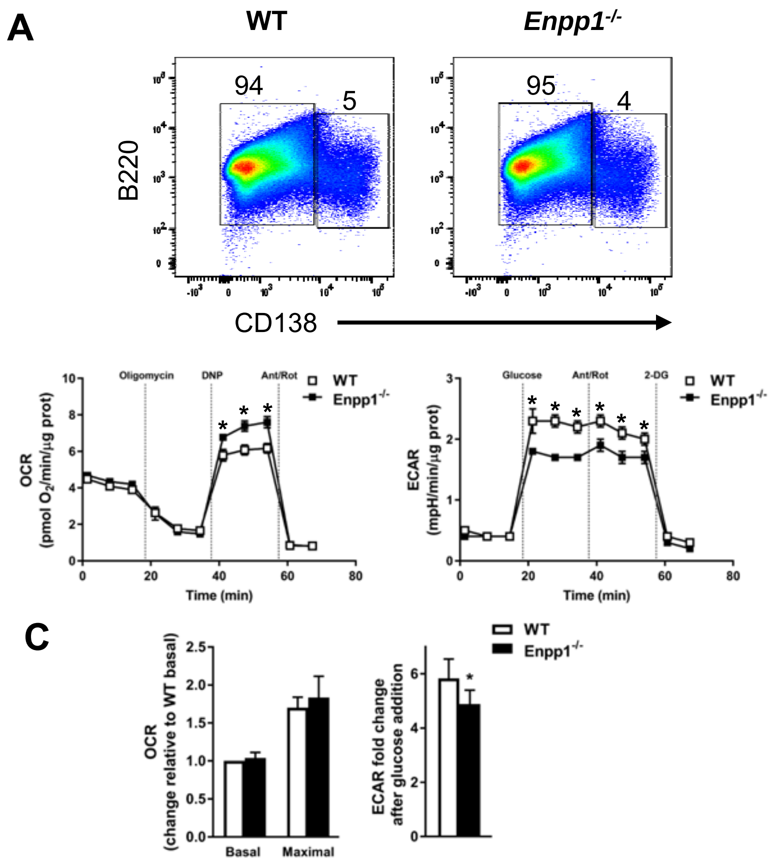

Figure S6. ENPP1-deficient PBs have reduced levels of glycolysis than wild-type PBs. (A) Purified B cells were stimulated with LPS (20 μg/ml) for 3 days to induce PB and PC development. The cells were stained with antibodies against B220 and CD138. The numbers are frequencies of cells falling in each gate. (B) Mitochondrial stress test to measure OCR (left panel) and glycolysis stress test to measure ECAR (right panel) of LPS-induced PBs. Error bars are technical replicates. *$p<0.05$. (C) Summary of relative basal and maximal respiration rate in response to DNP (left panel), and relative glycolysis rate after glucose addition to glucose-starved cells (right panel). Data are means ± SEM of 4 independent experiments. *$p<0.05$.
